# Interfamily variation in amphibian early life-history traits: raw material for natural selection?

**DOI:** 10.1002/ece3.287

**Published:** 2012-07

**Authors:** Gareth R Hopkins, Brian G Gall, Susannah S French, Edmund D Brodie

**Affiliations:** 1 Department of Biology, Utah State University5305 Old Main Hill, Logan, Utah 84322; 2 The Ecology Center, Utah State University5305 Old Main Hill, Logan, Utah 84322

**Keywords:** Amphibian, egg, embryonic development, hatching, Salamandridae, *Taricha granulosa*, variation

## Abstract

The embryonic development and time to hatching of eggs can be highly adaptive in some species, and thus under selective pressure. In this study, we examined the underlying interfamily variation in hatching timing and embryonic development in a population of an oviparous amphibian, the rough-skinned newt (*Taricha granulosa*). We found significant, high variability in degree of embryonic development and hatching timing among eggs from different females. Patterns of variation were present regardless of temperature. We also could not explain the differences among families by morphological traits of the females or their eggs. This study suggests that the variation necessary for natural selection to act upon is present in the early life history of this amphibian.

## Introduction

Some of the earliest traits subject to natural selection in an oviparous animal's life are those concerning embryonic development and the timing of the first life-history switch point, when eggs hatch (reviewed by Warkentin 2011). While timing of hatching is often thought to be highly canalized, this has shown to not always be the case in a large variety of taxa, and changes in hatching timing and rates of development can have great effects on the fitness of these organisms (reviewed by Warkentin 2011). For example, variation in the timing of egg hatching in damselflies may allow some individuals to persist while others die in ephemeral habitats where hydroperiod is in constant flux (De Block et al. 2005), and hatching timing in monogenean parasitic worms which infect swimming fish is crucial to facilitating successful host infection (Whittington and Kearn 2011). In amphibians, red-eyed tree frogs (*Agalychnis callidryas*) hatch early to avoid predation by egg-eating snakes and wasps, but this also results in individuals hatching at a smaller size and developmental stage, and thus being more vulnerable to larval predators (Warkentin 1995, 1999, 2011). The timing and size at which *Ambystoma opacum* and *Ambystoma talpoideum* salamanders hatch influences their survival and interspecific competitive and predatory interactions as larvae (Boone et al. 2002), and larvae of the rough-skinned newt, *Taricha granulosa*, that are smaller and less developed are more likely to die from predatory attacks by dragonfly nymphs (Gall et al. 2011a). Thus, the rate and degree of embryonic development that occurs in the egg capsule, and the timing of hatching from that capsule are clearly highly adaptive traits that can have considerable influence on performance during later life stages. However, while clearly adaptive, it is still unclear if these early life-history traits are subject to natural selection, as we have little knowledge of how they vary within a population.

Variation, regardless of its cause, is the underlying raw material for natural selection. If that variation is heritable, it can lead to evolutionary change. Therefore, although rarely completely possible to do, when trying to understand the potential for evolution, it is important to try to distinguish between the different potential sources of phenotypic variation (Berven 1982; Travis 1983; Laugen et al. 2005).

Temperature is an abiotic environmental influence that can have profound impacts on the embryonic development of amphibians (Bachmann 1969; Brown 1975; Bradford 1990; Voss 1993). Warmer temperatures cause animals to hatch sooner, but at less developed stages than animals raised in colder temperatures (Brown 1975; Williamson and Bull 1989; Voss 1993). We have little knowledge on how these differing environmental temperatures might impact any underlying variation present within a population. There is also a large body of literature which demonstrates that maternal effects such as female and egg size can greatly influence embryonic development and hatching timing (e.g., Kaplan 1980; Crump 1984; Semlitsch and Gibbons 1990; Semlitsch and Schmiedehausen 1994). However, not all studies on these early life-history traits have accounted for maternal effects, as maternal identity is often unknown (e.g., Thumm and Mahony 2002).

**Figure 1:**
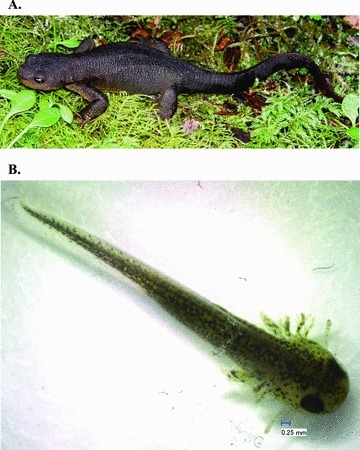
The study organism, *Taricha granulosa*. (A) Adult newt. (B) Newly hatched larva, developmental stage = 42.

The rough-skinned newt (*T. granulosa,* Skilton; Caudata: Salamandridae) (Fig. 1) is an amphibian species that is well suited to determining the interfamily variation that may be present in embryonic development and hatching timing. Gravid female newts are easily collected and can be induced to deposit their eggs in the laboratory. Thus, the maternal source of each egg is known, the environment in which the eggs are raised can be controlled, and some morphological characteristics of the female (e.g., size, weight, egg diameter) that might influence embryonic development and hatching timing can be measured and accounted for.

The purpose of this study was to determine if the highly adaptive traits of embryonic development and hatching timing varied significantly among the eggs from different *T. granulosa* females from a single population (which we hereafter refer to as “families”). As variation is the basis for natural selection, establishing if this variation exists is critical for future studies on the evolutionary biology of amphibian early life-history stages. To achieve our goal, we set out to determine if there was underlying variation in hatching timing, developmental stage, and size at hatching among the newt families, and if this variation was present at different environmental temperatures. As the majority of studies on amphibian early life-history traits have not considered differences among families in any of these traits, we used this consensus view in the literature to establish a testable null hypothesis: that there is no variation in hatching timing and embryonic development among newt families from a single population.

## Materials and Methods

### Experimental animals

A total of 27 gravid, adult female *T. granulosa* were collected from Soap Creek ponds in Benton County, Oregon, in 2010 and 2011. These ponds represent a homogenous environment. Animals were transported back to Utah State University and held individually in plastic containers (35 × 20 × 13 cm) with 3.5 L of filtered, chilled tap water. Newts were housed in environmental control chambers at 14°C (2010) or 7°C (2011) and fed blackworms (*Lumbriculus variegatus*) ad libitum.

Each female was injected with 10 μl luteinizing hormone releasing hormone ([des-Gly10, D-His(Bzl)6]-LHRH ethylamide; Sigma #L2761, Sigma Aldrich, St. Louis, MO) to induce egg deposition and provided a small piece of polyester fiber as an oviposition site. Eggs were collected and removed within 12 h of deposition, at which point timing of the length of the embryonic period began. After all eggs were deposited, the mass and snout-vent length (SVL) of each female was recorded. For eggs raised at 7°C, the mean egg diameter for each female also was calculated by recording the egg diameter for 10 eggs per female (eggs not used in the experiment) using an ocular micrometer with an Olympus stereo microscope.

### Embryonic development at 14°C

Eggs from 11 female newts were placed into 3.5-cm diameter round numbered cups with 2 mL of filtered tap water in groups of five in March 2010. In total, 444 cups were filled with 2220 eggs. The total number of eggs used from an individual female ranged from 110 to 285.

Cups were placed on shelves in an environmental control chamber at 14°C and were monitored for hatching at 0700 and 1900 h. When a larva had completely hatched and was free-swimming (evident by straightening of the body), the larva was removed from the cup with a pipette, and the time to hatching and developmental stage were recorded. Each larva was photographed (Nikon™ D70 digital camera with a 150 mm micro lens) to determine total length at hatching which was calculated from photos using ImageJ (U.S. National Institutes of Health, Bethesda, MD).

We utilized the standard salamander early life-history staging protocol by Harrison (1969), using an Olympus stereo–microscope to examine each hatchling for the presence of diagnostic morphologies particular to each stage. This is a 46-stage diagnostic table, and all eggs used at both temperatures hatched between stages 39 and 43. Stage-specific morphologies include the emergence, length, and shape of gills; limb-buds; balancers; eyes; mouth; etc. (see Table 1 for more detail).

**Table 1:** Diagnostic characteristics used to determine developmental stages at hatching in *T. granulosa* hatchlings in this study. Stages encountered include stages 39–43 from Harrison's (1969) 46 stage standard salamander staging table. Descriptions of these stages are reprinted below

Stage	Description
39	Gills reach forelimb bud, balancer club-shaped
40	Gills curved dorsally, forelimb bud flattened distally, pigmentation of iris visible
41	Forelimb bud notched distally
42	Forelimb with deeper bifurcation distally and slight bulge marking beginning of elbow joint
43	Mouth opens

In this experiment we also exposed developing newt eggs to filtered tap water conditioned with chemical stimuli from one of seven treatments simulating the presence of egg or larval predators; however, chemical stimuli had no effect on the results (main effect or interaction with individual female) and thus will not be discussed further.

### Embryonic development at 7°C

Eggs from 16 female newts were placed into cups with 2 mL of 20% Holtfreter's solution (a medium recommended for the successful development of caudate embryos (Armstrong et al. 1989)) in groups of three in April 2011. Eggs were raised in an environmental control chamber in a similar manner to the 2010 experiment, except at 7°C instead of 14°C. In total, 842 cups were filled with 2526 eggs. The total number of eggs used from an individual female ranged from 60 to 294, equating to 20–98 cups (mean ± SE number of cups per female = 52.6 ± 6.3). Cups were checked daily for dehydration, and distilled water was added if needed to maintain a constant water level in each cup. Cups were checked daily for hatched eggs, at which time the larvae were removed, staged, and their length measured using an ocular micrometer with an Olympus stereomicroscope.

### Statistical analyses

We examined the effect of individual female on the hatching time, developmental stage, and total length of recently hatched newt eggs using a general linear model ANOVA with female set as a random factor. Time to hatching, developmental stage, and total length at hatching were analyzed with a normal distribution with the identity link function. All variables met model assumptions of normality and homo–scedasticity. Cups were considered replicating units, with eggs within each cup incorporated into the model as sub–samples, by using the average value of all eggs within each cup as the unit of analysis.

If individual female was found to have a significant effect on any of the response variables, we conducted simple linear regressions of female mass and SVL (both temperatures) and mean egg diameter and total number of eggs (7°C) on the response variables to see if these traits could explain the variation among families.

All statistical analyses were conducted using SAS software Version 9.2 (SAS Institute Inc., Cary, NC).

## Results

### Embryonic development at 14°C

**Figure 2:**
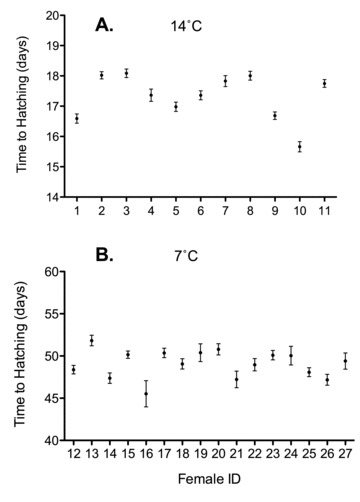
Significant variation in hatching timing among eggs of different female *Taricha granulosa* from a single population. (A) Results (mean ± SE number of days) for the 11 females whose eggs were raised at 14°C. (B) Results (mean ± SE number of days) for the 16 females whose eggs were raised at 7°C.

**Figure 3:**
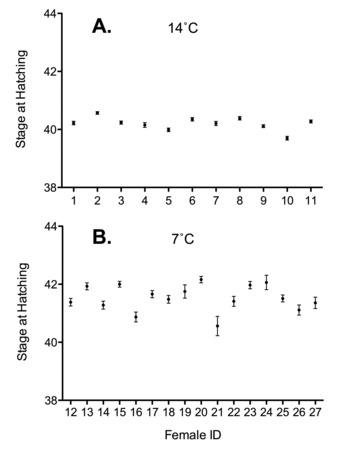
Significant variation in developmental stage at hatching among hatchlings of different female *Taricha granulosa* from a single population. (A) Results (mean stage ± SE) for the 11 females whose eggs were raised at 14°C. (B) Results (mean stage ± SE) for the 16 females whose eggs were raised at 7°C.

**Figure 4:**
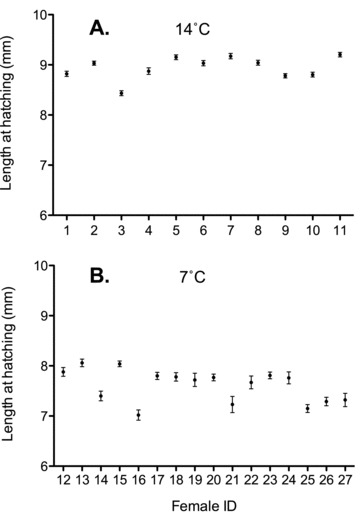
Significant variation in size (total length in mm) at hatching among hatchlings of different female *Taricha granulosa* from a single population. (A) Results (mean ± SE) for the 11 females whose eggs were raised at 14°C. (B) Results (mean ± SE) for the 16 females whose eggs were raised at 7°C.

There was significant variation (all *P* < 0.0001, Table 2) among females in life-history characteristics resulting in some females’ offspring hatching more than two days before others (Fig. 2A), almost a full developmental stage earlier (Fig. 3A) and 0.8 mm smaller (Fig. 4A).

**Table 2:** Statistical results of the effect of individual female on time to hatching (days), stage at hatching, and size (length, mm) at hatching for *T. granulosa* embryos raised at 14°C and 7°C

	14°C						7°C					
		
	*N*	Mean (SD)	*F*	MS (model, error)	df (*n*,*d*)	*P*	*N*	Mean (SD)	*F*	MS (model, error)	df (*n*,*d*)	*P*
Time to hatching	444	17.37 (1.23)	20.97	21.35, 1.02	11, 432	< 0.0001	842	49.40 (5.24)	4.92	126.28, 25.68	15, 826	< 0.0001
Stage at hatching	444	40.23 (0.48)	9.98	1.89, 0.19	11, 432	< 0.0001	841	41.65 (1.11)	7.37	8.14, 1.10	15, 825	< 0.0001
Size at hatching	444	8.94 (0.40)	21.81	2.13, 0.11	10, 359	< 0.0001	842	7.67 (0.72)	12.29	5.26, 0.43	15, 826	< 0.0001

There were no biologically meaningful (i.e., strong) relationships between the response variables and female SVL or mass. Although some regressions produced statistically significant results, no *R*^2^ value was greater than 0.07, with the majority being much smaller (mean ± SE [*R*^2^] = 0.02 ± 0.001); this indicates that female SVL and mass explained very little of the variation we observed in time to hatching, stage at hatching, and length at hatching.

### Embryonic development at 7°C

Mean time to hatching varied by nearly seven days (Fig. 2B), with some families hatching nearly two developmental stages earlier (Fig. 3B) and 1 mm smaller (Fig. 4B) on average than conspecific families. There was a significant effect of individual female on time to hatching, stage at hatching, and size at hatching (all *P* < 0.0001, Table 2).

No biologically meaningful relationships were evident between the response variables and female SVL, mass, mean egg diameter, or number of eggs laid. Although some regressions produced statistically significant results, no *R*^2^ value was greater than 0.06, with the majority being much smaller (mean *R*^2^ = 0.02 ± 0.004); this indicates that, like at 14°C, any measured female characteristic explained very little of the variation we observed in time to hatching, stage at hatching, and length at hatching.

### Temperature effects

Eggs took longer to hatch at 7°C than at 14°C (mean = 46.6 days vs. 17.4 days) (Fig. 2). Hatchlings raised at 7°C were smaller, but further developed, than hatchlings reared at 14°C (Figs. 3 and 4). Although interfamily variation was highly significant at both temperatures (all *P* < 0.0001, Table 2), the amount of variation at 7°C appeared to be greater overall than at 14°C (average range in time to hatching = 2.4 days at 14°C vs. 6.3 days at 7°C; stage at hatching = 0.87 vs. 1.6; size at hatching = 0.77 vs. 1.04). There was a significant difference in the variances of the two sets of females for time to hatching (*F*_15,10_ = 4.731, *P* = 0.017) and stage at hatching (*F*_15,10_ = 3.941, *P* = 0.034), but not size at hatching (*F*_15,10_ = 2.031, *P* = 0.260).

## Discussion

The present study demonstrates, with 27 different females and 4746 individual eggs, significant variability in embryonic development and time to hatching among families of *T. granulosa* from a single population, and that these patterns of variation are present regardless of environmental temperature. We could not explain differences among families by female morphology or egg or clutch size.

We found a slight decrease in variability at higher temperatures, but the overall effect of family on all three response variables (i.e., hatching timing, developmental stage, size at hatching) was strong for both temperatures. Consistent with these results, other studies have also found that there is less variability in hatching timing for individuals hatching at high versus low temperatures (Voss 1993). The effect of temperature, a key environmental influence, on amphibian embryonic development, is well known (Bachmann 1969; Brown 1975; Bradford 1990; Voss 1993), and our results are consistent with previous studies. Newt embryos reared at 14°C hatched 41.5 days sooner than those raised at 7°C, but at an earlier developmental stage, as has been found in other amphibians (Brown 1975; Williamson and Bull 1989; Voss 1993). A similar study on the sympatric salamander *Ambystoma gracile* found a difference of 62 days in the hatching timing of eggs raised at 7°C versus 12°C (Brown 1975).

Maternal effects on the phenotype of her offspring are ubiquitous among both plants and animals, especially in relation to life-history traits (reviewed by Räsänen and Kruuk 2007). In our study, we considered two easily measurable sources of potential maternal effects that could influence early life-history traits of newts: maternal size (SVL and weight) and mean egg diameter. We found no biologically significant effect of either trait on hatching timing, developmental stage, or size at hatching in *T. granulosa*. Female (Travis 1983; Semlitsch and Schmiedehausen 1994; Crespi and Lessig 2004) and egg sizes (Kaplan 1980; Crump 1984; Kaplan 1985, 1989; Semlitsch and Gibbons 1990; Crespi and Lessig 2004; Thumm and Mahony 2005; Kaplan and Phillips 2006) significantly influence hatching timing and embryonic development in other amphibian species. However, some experiments have found mixed influences of maternal effects, with either female body size and/or egg diameter significantly correlating with some offspring life-history traits but not with others (Kaplan 1980; Travis 1983; Travis et al. 1987; Kaplan 1989; Thumm and Mahony 2005). Still other studies found that maternal effects play little or no role in explaining significant interfamily variation in various life-history traits including hatching timing and embryonic development; in these studies genetic effects explained more of the variation (Williamson and Bull 1989; Newman 1994; Kopp and Baur 2000; Laurila et al. 2002; Alcobendas et al. 2004; Laugen et al. 2005). While many studies have found that nongenetic maternal effects explain very little variation in developmental traits, and our analyses seem to support this assertion, we cannot absolutely discount other unmeasured maternal effects, such as female lipid, hormone content, or yolk quality (e.g., Crump and Kaplan 1979) from playing a role in explaining the variation we observed.

The few studies that have successfully partitioned out the genetic nature of variability in amphibians from maternal and environmental influences have found a significant underlying genetic basis for variability in larval and juvenile life-history traits (Berven 1982; Travis et al. 1987; Newman 1994; Kopp and Baur 2000; Alcobendas et al. 2004; Laugen et al. 2005). Our results that variation among families persisted regardless of environmental temperature or measured maternal effects suggests that the variation we observed in these traits may be due to genetic differences between individual females. However, more data, including paternal identity, is needed to definitively determine if the variation we observed in hatching timing and embryonic development is genetic, and thus able to lead to evolutionary change. Regardless of its exact cause, however, the interfamily variation we observed in this study is substantial, and it is this variation that can serve as the raw material for natural selection.

For natural selection to act upon a trait, there must not only be significant variation in the trait within a population, but such variation must also confer survival advantages to some individuals but not others. The traits we examined for interfamily variation in this study are all highly adaptive. Embryonic development and hatching timing may considerably influence an individual's performance during later life stages in both invertebrates and vertebrates (De Block et al. 2005; Warkentin 2011; Whittington and Kearn 2011). In amphibians, hatching timing, size, and stage have been shown to have significant fitness consequences in both anurans (Warkentin 1995, 1999) and caudates (Boone et al. 2002) in relation to survival, the onset of feeding, and competitive and predatory interactions. The survival of *T. granulosa* larvae in particular, in predatory encounters with dragonfly nymphs, is affected by the size of the larva (Gall et al. 2011a). In addition, at the population we studied, larval caddisflies (Trichoptera) are a major predator on *T. granulosa* eggs, and have the potential to eliminate the entire reproductive output of the newt population in as little as 36 h (Gall et al. 2011b). The presence of interfamily variation in size, developmental stage, and time to hatching may lead to differential survival between clutches, and therefore be critically important for the evolution of rapid development in response to selection from this predator.
